# Ultrasonic activation of calcium silicate sealers in single-cone obturation: A micro-CT study of voids and gaps formation

**DOI:** 10.1371/journal.pone.0356065

**Published:** 2026-08-14

**Authors:** Ammar AbuMostafa, Maram AlHati, Mohammad Alabdulkareem

**Affiliations:** 1 Riyadh Elm University, College of Medicine and Dentistry, Department of Restorative Dentistry, Kingdom of Saudi Arabia, Riyadh, Saudi Arabia; 2 Private Practice, Kingdom of Saudi Arabia, Riyadh, Saudi Arabia; Universidade Federal Fluminense, BRAZIL

## Abstract

**Aim:**

This study investigated the effect of ultrasonic activation (UA) on porosity formation in root fillings using two calcium silicate-based sealers.

**Methods:**

Forty single-rooted premolars were decoronated, and the roots were adjusted to a standardized length of 11 mm. Canals were shaped with the #F3 ProTaper Ultimate file system. Teeth were divided into two groups for single-cone obturation with two different sealers: Angelus Bio-C Sealer (n = 20) and TotalFill BC Sealer (n = 20). Each group was subdivided into two subgroups (with and without UA). In the UA groups, the tweezers holding the GP cone were contacted by a smooth ultrasonic tip for two to three seconds. After obturation, samples were scanned using micro-CT. Group differences in mean total porosity were analyzed with Mann-Whitney Z and Kruskal-Wallis tests, followed by Dunn-Bonferroni post-hoc tests. Interfacial gaps and internal voids were also quantified separately to assess distribution.

**Results:**

TotalFill BC Sealer with ultrasonic activation demonstrated the highest mean total porosity, whereas Angelus Bio-C Sealer without ultrasonic activation exhibited the lowest, though this difference was not statistically significant (p  =  0.428). Quantitative analysis revealed that interfacial gaps were significantly higher than internal voids across all groups (p < 0.05). Total porosity varied significantly across root levels (p < 0.001), with the cervical third showing the greatest proportion of porosities, followed by the apical third, while the middle third consistently demonstrated the lowest porosity volume.

**Conclusion:**

No significant difference in total porosity was found between Bio-C Sealer and TotalFill BC Sealer, regardless of ultrasonic activation. However, interfacial gaps represented the majority of the total porosity volume, and significant differences in distribution were observed across different root levels.

## Introduction

An ideal root canal filling technique should entirely and uniformly fill the canal without any voids throughout its length, ensuring a proper apical seal [1]. The presence of voids in the filling can create pathways for leakage. The critical role of obturation influences treatment outcomes, as a proper seal is essential to achieve long-term success of the root canal system [2,3].

As a substitute for the continuous wave technique, which depends on epoxy resin-based sealers, the single-cone obturation approach employing calcium silicate sealers, has been recommended [4]. More voids have been reported in the cervical third when using the single-cone technique without apical pressure compared with the continuous-wave technique [5]. Therefore, it is important to explore methods, such as ultrasonic activation, that can enhance the quality of filling with these sealers.

Ultrasonic activation improves the sealer's penetration into complex areas, reaching deeper into dentinal tubules with fewer gaps [6–7]. The activation occurs using specific ultrasonic tips that are connected to devices producing high-frequency vibrations (25–30 kHz), which promote acoustic transmission and cavitation [8].

### Aim

This study seeks to determine how ultrasonic activation affects void formation in the single cone obturation technique when using Angelus Bio-C Sealer and TotalFill BC Sealer. The rationale stems from concerns that voids may compromise the canal seal. Determining whether activation reduces voids carries clinical significance for improving long-term endodontic outcomes.

### Null Hypothesis

There is no significant difference in the amount of voids formed in root canals filled with Angelus Bio-C Sealer or TotalFill BC Sealer when using the single cone obturation technique, whether ultrasonic activation is applied or not.

## Materials and methods

### Ethical approval

This study was reviewed and approved by the Institutional Review Board (IRB) of Riyadh Elm University. The approved protocol was titled “Effects of Ultrasonic Activation of Calcium Silicate–Based Sealers on Root Canal Filling Quality Using Single Cone Obturation – An in vitro study” and was granted ethical clearance under IRB approval number FPGRP/2024/847/1115/1020. Consent forms were exempted from informed consent, it is a laboratory – based study with no involvement of human subjects, extracted teeth were extracted for Orthodontic or Periodontic reasons and not for the purpose of this study. The waiver of informed consent was approved by the same Institutional Review Board. Teeth collected in the dental clinics of the university hospital.

### Data access for research purposes

The study data were accessed for research and analysis purposes between 1/1/2025 and 1/2/2025.

### Sample size

Based on the void volume means and standard deviations reported by Celikten et al. for four different sealers in the coronal third (means 0.818–1.564; common SD ≈ 0.484), the effect size for a one-way fixed-effects ANOVA with four groups was calculated in G*Power (version 3.1) as f ≈ 0.69, indicating a medium-to-large between-group effect [9]. Using this effect size with α = 0.05 and desired power of 90% (1–β = 0.90), the a priori power analysis yielded a minimum required total sample size of 36 roots (9 specimens per group). To compensate for an anticipated 10% rate of non-response or unusable specimens (e.g., fractures, imaging artefacts, or exclusions during preparation), the target sample was inflated to 40 roots, resulting in 10 teeth per group. This final sample size preserves power ≥90% to detect clinically meaningful differences in void volume between the experimental groups [10].

### Inclusion criteria

Teeth chosen for this study were without previous root canal treatment. Teeth were deemed ineligible if they displayed any cracks, perforations, signs of internal or external resorption, or carious lesions involving the root. All teeth were Vertucci Type I classification.

### Study groups

Teeth were equally distributed in 4 groups (n = 10/group), with ultrasonic activation (UA) and without:

- Angelus Bio-C sealer with UA.
- Totalfill FKG sealer with UA.
- Angelus Bio-C sealer without UA.
- Totalfill FKG sealer without UA.

### Sample preparation

40 Single-rooted teeth (mandibular premolars) extracted for orthodontic purposes were selected. A universal curette (M 23, Deppeler, Rolle, Switzerland) was utilized to scale the root surfaces, eliminating soft tissue, calculus, and bone deposits. Following scaling, each tooth underwent disinfection through immersion in 5.25% sodium hypochlorite (NaOCl) for two hours. Subsequently, the specimens were preserved in 0.1% Thymol solution until testing commenced. Before selection, extracted teeth underwent preoperative radiographic evaluation to examine their internal anatomy. Each tooth was examined under an optical microscope (OPMI pico; Zeiss Co., Jena, Germany) to confirm the presence of a single canal; specimens with multiple canals were excluded. For the selected teeth, the crowns were sectioned at the cemento-enamel junction, and the roots were cut to a uniform length of 11 mm. Canal patency was established by inserting a #10 K-file (Dentsply Maillefer, Ballaigues, Switzerland) into each canal until the tip was visible at the apical foramen. The working length was then determined by subtracting 0.5 mm from this measurement. Root canal preparation was carried out using an Endo Radar Plus motor (Woodpecker, Guilin, China) and the ProTaper Ultimate™ system (Dentsply Sirona, Ballaigues, Switzerland), following the manufacturer's instructions precisely. Instrumentation proceeded sequentially, beginning with the SX file followed by the S1 shaping file, both operated at a torque of 3 Ncm. Next, the S2 shaping file was used at a torque of 1 Ncm. This was followed by the F1 finishing file at a torque of 1.5 Ncm, and finally the F2 and F3 finishing files, both operated at a torque of 2.0 Ncm. All files were used at a constant speed of 250 rpm. During instrumentation, 2.5% sodium hypochlorite (NaOCl) was used as the irrigant. A final irrigation sequence was then performed, which involved applying 2 mL of 2.5% NaOCl, followed by 2 mL of 17% EDTA (Patterson Dental Supply, Dallas, TX) for one minute, and concluding with 10 mL of distilled water. Following the irrigation steps, the canals were dried using paper points (Dentsply Tulsa Dental, Johnson City, TN). Canal preparation was standardized to an F3 size (apical size 30) to obtain a consistent apical diameter that supports effective irrigant penetration and improves obturation quality, as demonstrated in studies on mandibular premolar morphology and on the impact of apical preparation size on irrigant penetration and obturation quality [11,12].

### Root canal filling

**Fig 1:**
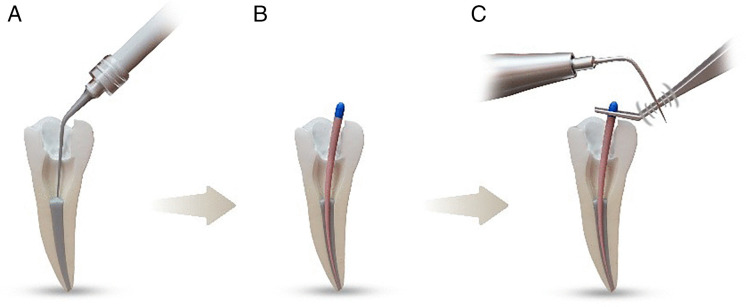
Illustration of the experimental obturation procedure and indirect ultrasonic activation method. (A) The calcium silicate-based sealer was introduced into the prepared root canal using the intracanal tip provided by the manufacturer, ensuring passive delivery. (B) A corresponding gutta-percha cone was then inserted to the full working length following the single-cone obturation technique. (C) Indirect ultrasonic activation was achieved by bringing a smooth ultrasonic tip into contact with the cotton pliers holding the gutta-percha cone. Activation lasted 2–3 seconds, enabling ultrasonic energy transfer to the sealer without direct contact between the tip and the canal interior.

An investigator who was not involved in the allocation process and remained blinded to it randomly divided the specimens into four experimental groups, each containing ten teeth. The two root canal sealers were prepared following the respective manufacturers’ directions. All teeth were then obturated using the single-cone technique with matching ProTaper Gutta-Percha F3 cones (Dentsply Sirona, Ballaigues, Switzerland), according to the following steps:

- Each sealer was placed passively into the canal using its dedicated intra-canal tip. The tip was positioned approximately 2–3 mm from the working length to allow the sealer to flow apically in a controlled manner without applied pressure, which helped achieve a consistent coating of the canal walls.
- After the sealer and gutta-percha (GP) cone were positioned, a smooth ultrasonic tip (StartX #3, Dentsply Maillefer) was placed in contact with the cotton pliers holding the GP cone. The P5 Newtron® ultrasonic unit (Satelec Acteon, Merignac, France) was then activated at power level “8” for two to three seconds, in line with the manufacturer's recommendations. The activation continued until the GP cone reached the full working length. An overview of the obturation process and the indirect ultrasonic activation method is shown schematically in Fig 1.
- System B heat source equipped with a medium-sized heated plugger tip (SybronEndo, Orange, CA, USA) was used to remove excess gutta-percha at the canal orifice level.

For the non-ultrasonic activation groups, the identical protocol was followed, except that the ultrasonic device was not activated. After obturation was complete, all specimens were stored in 0.1% Thymol solution at 37°C and 100% humidity for five days to ensure the sealer had fully set. A second investigator, also blinded to the group assignments, carried out the random selection of samples for subsequent analysis.

### Micro-CT evaluation

For micro-CT scanning, each specimen was positioned inside the imaging chamber so that its long axis was perpendicular to the direction of the X-ray beam. A Bruker SkyScan 1172 high-resolution micro-CT system (Bruker SkyScan, Kontich, Belgium) was used for all scans. The following standardized acquisition parameters were applied: 93 kV voltage, 80 μA anode current, 885 ms exposure time, 12 μm pixel size, and a 0.4° rotation step over a full 360° scan. To enhance signal quality, frame averaging was set to 4, and a random movement of 8 was used to reduce ring artifacts. A copper and aluminum filter was also placed in the beam path to minimize beam-hardening effects. Additionally, a flat-field correction was performed to account for any differences in sensitivity among individual camera pixels.

Following acquisition, the scanned projections were reconstructed into cross-sectional images using N-Recon software (version 1.6.9.4, Bruker SkyScan, Kontich, Belgium). Image optimization settings were reviewed and configured appropriately prior to reconstruction. To address background inconsistencies detected by the X‑ray camera, ring artifact reduction was applied at a level of 5. A beam hardening correction of 25% was used to prevent artificial density differences between the outer and inner portions of each specimen. Smoothing was performed using a Gaussian filter with a kernel size of 2. Given the broad range of material densities present, all images were saved in 16‑bit TIF format.

All reconstructed datasets were subsequently imported into Dataviewer® software (version 1.5.6.2, Bruker SkyScan, Kontich, Belgium). This program was used for co-registering pre‑ and post‑scan datasets, evaluating image quality, reorienting and resizing images, and performing visual assessments of the three‑dimensional data. After co-registration, the datasets were transferred to CTAn® software (version 1.20.8.0) for further processing, which included image visualization, region‑specific analysis, binarization, and quantification of the root canal structures based on grayscale values. Finally, CTVol® software (version 2.3.2.0, Bruker SkyScan, Kontich, Belgium) was used to generate three‑dimensional reconstructions and produce color‑coded images of the specimens.

The following calculations were used to determine the volumetric parameters:

- Total Canal Volume = Filling Material Volume + Total Porosity Volume
- Total Porosity Volume = Interfacial Gaps (Open Porosity) + Internal Voids (Closed Porosity)
- Total Porosity Percentage = (Total Porosity Volume / Total Canal Volume) × 100

For clarity, the following operational definitions were applied:

- Total Canal Volume: The complete internal space bounded by the root canal walls.
- Filling Material Volume: The combined volume occupied by both gutta-percha and sealer.
- Interfacial Gaps (Open Porosity): Spaces located at the interface between the filling material and dentinal walls.
- Internal Voids (Closed Porosity): Spaces entirely enclosed within the filling material mass.
- Total Porosity: The combined volume of both interfacial gaps and internal voids.

## Results

Descriptive statistics, including mean, standard deviation, median, minimum, and maximum values, were calculated for the porosity volume measurements obtained from the Angelus Bio-C and TotalFill groups, both with and without ultrasonic activation. To evaluate differences in mean volume porosities among the experimental groups, the Mann-Whitney Z-test and Kruskal-Wallis test was applied. When statistically significant differences were found, post hoc comparisons were conducted using the Dunn-Bonferroni test. The Shapiro-Wilk test was used to evaluate the normality of the data distribution; the results confirmed a non-normal distribution, thereby supporting the use of nonparametric tests. A significance threshold of p < 0.05 was established for all statistical analyses. All statistical calculations were performed using SPSS software (Version 26, IBM Corporation, Armonk, NY, USA).

The highest mean percentage of of total porosity was observed in the TotalFill with UA group (0.53), followed by Angelus Bio-C with UA (0.37), TotalFill without UA (0.30), and finally Angelus Bio-C without UA (0.29). However, statistical analysis revealed no significant difference between these groups (p = 0.428) (Table 1).

**Table 1:** Distribution of total porosity percentage in each group (n = 40).

Group (n = 10)	Mean	SD	P-value ^§^
Angelus Bio-C with UA	0.37	0.31	0.428
TotalFilL with UA	0.53	0.44	
Angelus Bio-C without UA	0.29	0.22	
TotalFill without UA	0.30	0.23	

§ P-value has been calculated using Kruskal-Wallis test.

The volumetric analysis revealed that interfacial gaps (open porosities) accounted for a significantly higher percentage of the total porosity compared to internal voids (closed porosities) across all tested groups (p < 0.05) (Table 2).

**Table 2:** Comparison of the percentage volume of internal voids and interfacial gaps for each group.

Group (n = 10)	Internal Voids (Closed Porosity)		Interfacial Gaps (Open Porosity)		P-value ^§^
	Mean	SD	Mean	SD	
Angelus Bio-C with UA	0.09	0.05	0.28	0.15	<0.05
TotalFilL with UA	0.13	0.07	0.40	0.21	<0.05
Angelus Bio-C without UA	0.08	0.05	0.21	0.11	<0.05
TotalFill without UA	0.06	0.04	0.24	0.12	<0.05

§ P-value has been calculated using Kruskal-Wallis test.

**Fig 2:**
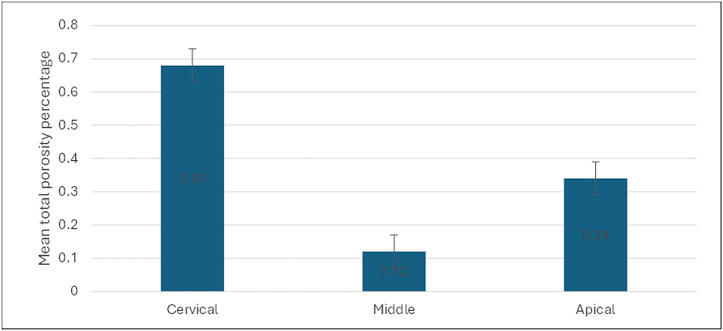
Comparison of porosity percentage according to location.

**Fig 3:**
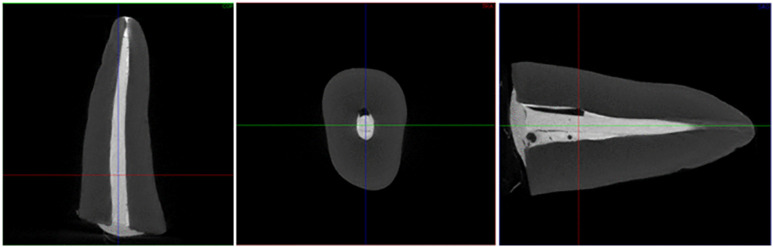
3D micro-CT images of the obturation in a sample from the Angelus Bio sealer with ultrasonic activation (UA) group. (3A) Sagittal view. (3B) Axial view. (3C) Coronal view.

**Fig 4:**
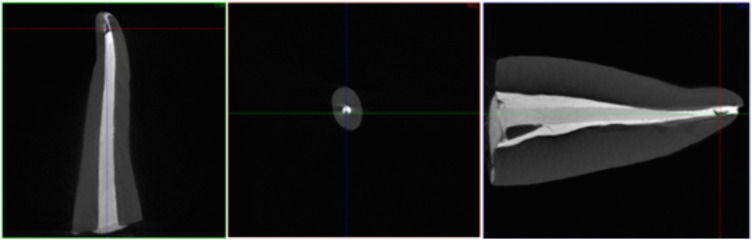
3D micro-CT images of the obturation in a sample from the TotalFill sealer with ultrasonic activation (UA) group. (4A) Sagittal view. (4B) Axial view. (4C) Coronal view.

**Fig 5:**
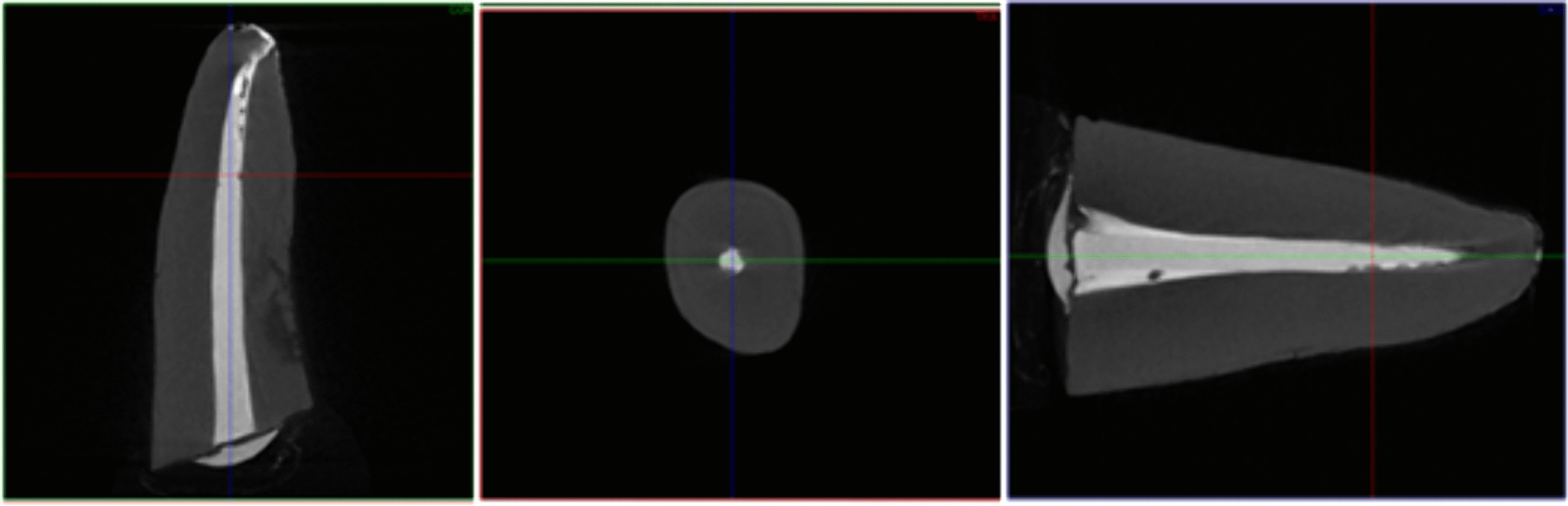
3D micro-CT images of the obturation in a sample from the TotalFill sealer without ultrasonic activation (non-UA) group. (5A) Sagittal view. (5B) Axial view. (5C) Coronal view.

For Angelus Bio-C with UA, the cervical third (0.78 ± 0.29%) showed a significantly greater total porosity percentage compared with both the middle (0.21 ± 0.03%) and apical (0.21 ± 0.04%) thirds, which did not differ from each other. For TotalFill with UA, the cervical third (1.12 ± 0.07%) also demonstrated a significantly higher total porosity percentage than the middle (0.18 ± 0.08%) and apical (0.29 ± 0.11%) thirds, which were statistically similar. In Angelus Bio-C without UA, all locations were significantly different, with the apical third (0.52 ± 0.03%) having the highest porosity percentage, followed by the cervical third (0.29 ± 0.19%), and then the middle third (0.06 ± 0.02%) with the lowest value. For TotalFill without UA, the cervical (0.50 ± 0.24%) and apical (0.36 ± 0.09%) thirds had statistically similar total porosity percentage, and both were significantly greater than the middle third (0.05 ± 0.02%). In total, the average porosity percentage in the cervical region (0.68 ± 0.37) was significantly higher than both the middle (0.12 ± 0.08) and apical (0.34 ± 0.14) regions (Fig 2). The middle region had the lowest average porosity percentage compared to the other two regions, and the apical region also had a significantly higher average porosity percentage than the middle region. (Table 3). Representative 3D micro-CT views of the obturation patterns for Angelus Bio-C with UA (Fig 3), TotalFill with UA (Fig 4), and TotalFill without UA (Fig 5) are shown to illustrate the typical distribution of porosities in sagittal, axial, and coronal planes.

**Table 3:** Comparison total porosity percentage in each location among the group (n = 40).

Group	Total porosity percentage		
	Cervical	Middle	Apical
Angelus Bio-C with UA	0.78 ± 0.29 ^a^	0.21 ± 0.03 ^b^	0.21 ± 0.04 ^b^
TotalFill with UA	1.12 ± 0.07 ^a^	0.18 ± 0.08 ^b^	0.29 ± 0.11 ^b^
Angelus Bio-C without UA	0.29 ± 0.19 ^a^	0.06 ± 0.02 ^b^	0.52 ± 0.03 ^c^
TotalFill without UA	0.50 ± 0.24 ^a^	0.05 ± 0.02 ^b^	0.36 ± 0.09 ^a^
Total	0.68 ± 0.37 ^a^	0.12 ± 0.08 ^b^	0.34 ± 0.14 ^c^

Different superscript letters in the same horizontal row indicate statistically significant differences between root thirds (Cervical, Middle, and Apical) for that specific group (p < 0.05). Values sharing the same superscript letter in a row have no significant difference. The “Total” row represents the aggregate mean volume porosity percentage and standard deviation of all experimental groups combined, categorized by root third.

## Discussion

The objective of this investigation was to assess the impact of ultrasonic activation on porosity volume in two calcium silicate-based sealers, i.e., Angelus Bio-C Sealer and Totalfill FKG, when applied with the single cone obturation technique. The null hypothesis was upheld, as the presence or absence of ultrasonic activation did not produce significant differences in porosity formation within root canals filled with these sealers. However, significant differences in porosity volumes were observed between root levels within each sealer group, with the cervical region showing the highest porosity volume and the middle region the lowest.

This distribution pattern aligns with micro-CT findings showing that the middle third exhibits fewer porosities because its taper and geometry allow more predictable gutta-percha adaptation and hydraulic sealer movement, whereas the coronal and apical thirds present greater anatomical irregularities that predispose them to gap formation [13,14].

Micro-CT allows high-resolution, three-dimensional evaluation of root canal fillings and has been widely used to quantify voids and gaps and differentiate obturation materials in experimental studies [13,14]. It detects porosities that cannot be reliably identified on two-dimensional radiographs and enables clear distinction between gutta-percha, sealer, and internal or external voids [13,14]. Variations in canal taper alter root mechanical behavior, including fracture resistance [15], and may also influence material adaptation during obturation. Micro-CT literature often distinguishes between open and closed porosities [16]. Open porosities refer to gaps that appear at the sealer-dentin interface and may interrupt the continuity of the seal [17,18]. Closed porosities, on the other hand, are voids that lie completely within the sealer mass and do not affect the interface, making them generally less critical to sealing performance [19].

In the present study, quantitative analysis showed that open porosity was significantly higher than closed porosity across all groups. Specifically, open porosity averaged approximately 75.5 percent of the total porosity volume, while closed porosity accounted for an average of almost 24.5 percent. This differentiation is crucial because the high prevalence of open gaps suggests a greater potential impact on clinical success. From a clinical standpoint, gaps at the sealer–dentin interface are more concerning due to their role in endodontic failure [17–19].

After reviewing the 3D visualization and production of color-coded images of the samples, it appeared that sections with higher porosity volumes were the ones having large open porosities, which were found mostly in the middle or apical sections.

Achieving a hermetic seal in endodontic treatment is a critical factor that significantly influences long-term clinical success. This seal acts as a barrier against bacterial penetration and possible reinfection within the root canal system. Given the anatomical complexity and irregularities of root canals, which can shelter residual microorganisms, precise and thorough obturation becomes essential. The contemporary single cone obturation approach employing calcium silicate–based sealers, however, may present certain challenges, particularly in managing oval-shaped canals. Consequently, mandibular premolars were selected for this study because their oval canal configuration in the coronal and middle thirds provides a closer simulation of actual clinical conditions.

The current study indicated that the method used to insert sealers can impact the creation of voids and gaps. Among the two different techniques analysed, and like previous studies [20,21] ultrasonic activation of the sealer did not prove to be successful in reducing the presence of voids and gaps. Conversely, our findings differ from those of Kim et al. [22], who reported a significantly lower total porosity volume when using ultrasonic activation. This discrepancy may be related to the initial insertion technique of the gutta-percha (GP) cone; while we placed the GP cone directly into the canal, Kim et al. employed a manual up-and-down motion during insertion. This dynamic ‘pumping’ action likely facilitates a more uniform sealer distribution and helps displace trapped air before activation. The absence of this movement in our study may have resulted in a higher baseline of interfacial gaps that subsequent indirect activation could not fully overcome. Furthermore, the power settings and energy delivery differed between the studies. While Kim et al. used the lowest setting on an Obtura-Spartan unit, we utilized a ‘Power 8’ setting on the P5 Newtron device. Because ultrasonic frequencies and amplitudes are not standardized across manufacturers.

The higher mean total porosity percentage observed in the TotalFill with UA group, though not statistically significant, suggests that ultrasonic activation may have been counterproductive. It is hypothesized that the vibration could have introduced internal voids through air entrapment or created interfacial gaps by displacing the sealer unevenly along the canal walls.

The technique of obturation differs according to the manufacturer’s instructions from one type of CSBS sealer to another. Additionally, in our study itself, there was a difference between both types of sealers, where the Angelus Bio-C sealer had fewer porosities than the Totalfill, although not significant. Therefore, the type of sealer plays an important role in porosity formation. Although both sealers in our study had a similar composition, there’s a difference in their flowability and setting time.

When employing ultrasonic activation, it is advisable to use gentle vibrations, as excessive energy may alter the physical behavior of the material and negatively influence its handling characteristics [23]. In our study, we followed the methodology of a previous study [24], where they used the ultrasonic activation at the power of “8”, as indicated suitable for Endodontics by the manufacturer of the ultrasonic device. Currently, there are no established guidelines for the activation of sealers using sonic or ultrasonic methods. Therefore, additional research is essential to determine the possible advantages of these techniques regarding voids and gaps formation and clinical outcomes. None of the methods utilized in this study demonstrated areas free of gaps at the gutta-percha/sealer interface, aligning with findings from other studies [25].

Root canal anatomy is another factor that should be considered while evaluating porosity volume. It makes sense that more voids and gaps will occur in larger areas or areas with complex anatomy, than smaller areas. Understanding the morphological features and variations of root canals is crucial for successful treatment [26]. Approximately, 25% of teeth have oval or ribbon-shaped canals, making their preparation and filling particularly difficult [27]. The presence of accessory canals, isthmuses, and the complex three-dimensional network makes achieving a proper seal particularly challenging [28]. Furthermore, the intricate anatomy can harbour microbial biofilms, leading to persistent infections and treatment failures. Thus, understanding anatomical variations is crucial for endodontic success, and a thorough examination of both the techniques and materials used in treatment is necessary.

Micro-CT studies have provided additional insight into the present findings. One three-dimensional micro-CT investigation demonstrated that voids and gaps are consistently present within root canal fillings, with variations in their distribution across the coronal, middle, and apical thirds, regardless of the obturation material or technique used [29]. These findings are consistent with the present observation that ultrasonic activation does not eliminate porosity. Another investigation found no significant differences in total porosity volume among various sealer placement techniques, including sonic activation, indirect ultrasonic activation, syringe injection, and master cone coating alone [16]. Nevertheless, a trend toward reduced voids and gaps was observed when both sonic and ultrasonic activation were combined, although this approach was associated with increased apical extrusion of sealer, suggesting that activation may influence sealer behaviour without consistently improving internal adaptation.

Further research demonstrated that different calcium silicate-based sealers exhibit comparable filling percentages immediately after single cone obturation. However, all sealers showed a significant increase in porosity volume after aging in a phosphate rich environment, indicating that long-term material changes may play a larger role in porosity formation than the obturation technique itself [30]. Additional micro-CT analysis showed that all materials tested bioceramic, resin based, and glass ionomer-based sealers presented measurable porosities in oval canals [9]. While this study did not directly attribute porosity formation to canal size or irregularity, the findings support the influence of anatomical complexity on obturation quality, particularly in teeth with less circular canal shapes.

Beyond evaluating voids and gaps, another investigation reported that ultrasonic activation can enhance sealer penetration into dentinal tubules and may improve bond strength for certain sealers, although these improvements were not consistent across all materials tested [20]. Importantly, adhesive failures at the sealer–dentin interface remained prevalent, reinforcing the notion that activation alone may not overcome the intrinsic limitations of some sealers. Collectively, these studies support the present work in indicating that the effect of ultrasonic activation is variable and sealer dependent. Factors such as anatomical complexity, sealer composition, flow properties, and, as demonstrated in long-term studies, material aging, all contribute to the final obturation quality.

Indirect ultrasonic activation (UA) via tweezers was selected for its clinical advantages over direct intracanal methods. This approach is more time-efficient, requiring only 2–3 seconds of activation compared to the 20–40 seconds typically required for direct agitation with a spreader or ultrasonic tip [7,20,31]. It also enhances safety by eliminating risks of instrument fracture and iatrogenic damage, such as ledging or transportation, which can occur when vibrating metal tips contact apical dentinal walls. Furthermore, the indirect technique maintains a cleaner surgical field and better visibility by preventing sealer “splashing” caused by acoustic streaming. While these practical benefits dictated the methodology, the resulting energy attenuation through the tweezers and GP cone likely explains the lack of significant difference in porosity reduction, as the energy reaching the sealer was lower than that of direct-contact methods.

It’s important to have long term in vivo studies to assess the efficacy or ultrasonic activation in reducing voids and gaps overall the obturation. This includes trying the ultrasonic activation power in different intensities, different methods of sealer application, different obturation methods, different canal anatomy, and multirooted teeth.

In limitations, the research focused solely on Angelus Bio-C and Totalfill FKG sealers. Exploring a wider variety of Calcium Silicate Based Sealers (CSBS) could enhance insights into their different clinical performances in terms of porosity volume within the root canal, depending on the placement method used. Moreover, the porosity volume was assessed using only one analytical technique, specifically micro-CT imaging. It is important to interpret volumetric measurements from micro-CT carefully, as aspects such as voxel size, image processing software, and the radiopacity of materials might affect the outcomes. Another limitation is the different canal anatomy in each sample and the inability to standardize it in natural teeth. Therefore, voids and gaps could be larger in wide canals and smaller in narrower canals. Finally, the in-vitro setting doesn’t mimic the clinical scenario, and therefore it’s a limitation.

## Conclusion

Within the constraints of this investigation, the findings indicate that when calcium silicate-based sealers are used with the single cone obturation technique, the application of ultrasonic activation does not lead to a statistically significant reduction in void or gap volume within the root canal filling. Nevertheless, significant differences in porosity distribution were observed across the various root levels within each specimen. From a clinical perspective, the presence of voids and gaps in the root canal filling may compromise the seal by creating potential pathways for leakage, which could ultimately contribute to the failure of endodontic treatment.
